# Promising Option for Treatment of Striae Alba: Fractionated Microneedle Radiofrequency in Combination with Fractional Carbon Dioxide Laser

**DOI:** 10.1155/2016/2896345

**Published:** 2016-03-16

**Authors:** Farahnaz Fatemi Naeini, Shadi Behfar, Bahareh Abtahi-Naeini, Shima Keyvan, Mohsen Pourazizi

**Affiliations:** ^1^Skin Diseases and Leishmaniasis Research Center, Isfahan University of Medical Sciences, Isfahan, Iran; ^2^Cancer Research Center, Semnan University of Medical Sciences, Semnan, Iran; ^3^Department of Ophthalmology, Students' Research Committee, Isfahan University of Medical Sciences, Isfahan, Iran

## Abstract

*Background*. A consistent treatment has not been proposed for treatment of Striae Alba (SA). The present study was designed to compare the fractionated microneedle radiofrequency (FMR) alone and in combination with fractional carbon dioxide laser (FMR + CO_2_) in the treatment of SA.* Methods*. Forty-eight pairs of SA from six patients were selected. Right or left SAs were randomly assigned to one of the treatment groups. The surface area of the SA before and after treatment and clinical improvement using a four-point scale were measured at the baseline, after one and three months.* Results*. The mean age of the patients was 30.17 ± 5.19 years. The mean difference of the surface area between pre- and posttreatment in the FMR + CO_2_ group was significantly higher than that in the FMR group (*p* = 0.003). Clinical improvement scales showed significantly higher improvement in the FMR + CO_2_ group than in the FMR group in the first and second follow-up (*p* = 0.002 and 0.004, resp.). There were no major persistence side-effects in both groups.* Conclusions*. The results showed that FMR + CO_2_ laser was more effective than FMR alone in the treatment of SA.

## 1. Introduction

Striae distensae (SD), commonly known as stretch marks, represent linear dermal scars associated with epidermal atrophy [1, 2]. They are caused by progressive stretching of the skin connective tissue due to changes in contours of the body. These scars can be observed in the abdomen and breasts of pregnant women (striae gravidarum), bodybuilders, adolescents, and obese individuals [3, 4]. Disorders including Cushing's and Marfan's syndromes and prolonged steroid treatment are also associated with the development of SD [5, 6]. It is prevalent in all races; females are 2.5 times more susceptible than their male counterparts [3]. In the early stages, SD are pink or red lesions (striae rubra), which gradually mature and change into white wrinkled scars [striae alba (SA)] [4] and can lead to psychological stress [3, 4]. Several treatments have been advocated with variable efficacy. These include topical creams and even laser therapies; however, a consistent treatment has not been recognized to date. Various laser therapies are currently very popular [3]. Fractional photothermolysis, a laser treatment modality, offers collagen remodeling [3, 7, 8]; its efficacy has been confirmed with several studies [8–10]; however, the results are not adequate. In recent years, fractionated microneedle radiofrequency (FMR) device has been used in the treatment of atrophic scars and wrinkles and in skin rejuvenation [11–13]. This method is not only more effective but also with an acceptable safety profile [13–16].

Collagen remodeling is effected by the transfer of heat from the device to the dermal components including water, melanin, and collagen to release the secreting growth factor. This procedure involves the use of needles that can rupture blood vessels causing unwanted bleeding [17–20].

Recently, a study by Ryu et al. compared the efficacy of FMR alone and in combination with fractional carbon dioxide laser (FMR + CO_2_) in Korean patients with SD [17]. The positive therapeutic results of this study make it an important issue to be studied in other populations. To the best of our knowledge, a similar study has not been conducted in an Iranian population. The main purpose of our study was to compare the efficacy of FMR alone and FMR + CO_2_ for the management of distensae SA among Iranian patients.

## 2. Materials and Methods

### 2.1. Patients

Forty-eight pairs of lesions from six female Iranian patients were chosen by randomly selecting cases of SA from patients referred to the “Novin Laser Center” and “Sedigh-e-Tahereh Cutaneous Leishmaniasis Center,” Isfahan, Iran.

Inclusion criteria were the existence of SA on the abdomen, buttocks, flanks, and calves in skin type III. Exclusion criteria were pregnancy, breast feeding, striae on breasts and arms, Cushing's or Ehlers-Danlos syndrome, propensity for keloid formation, active infection in the treatment area, pacemaker implantation, isotretinoin use, filler injection, dermabrasion, or laser skin resurfacing in the past 6–12 months to the striae. The registration code of this study in the Iranian Registry of Clinical Trials (http://www.irct.ir/) is IRCT2014101519543N1, and the Ethical Committee of the Isfahan University of Medical Sciences approved the study protocol (project number: 393433). All the participants signed the written informed consent form.

**Figure 1:**
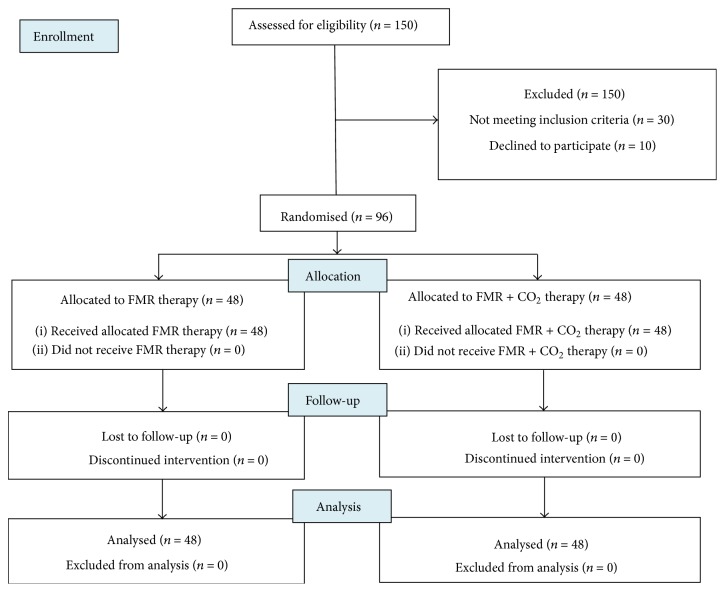
CONSORT flow chart of the study.

The flow chart of study is shown in Figure 1.

### 2.2. Laser Treatment

In each patient, pairs of striae with similar shape, size, and position were chosen from the right and left halves of the body. Following this, the right or left striae were randomly assigned to one of the treatment groups: FMR or FMR + CO_2_ group.

The FMR + CO_2_ group underwent one session of fractional CO_2_ laser, followed by three sessions of FMR and one more session of FMR + CO_2_ laser (overall, five sessions with four-week intervals). In contrast, the FMR group only underwent three sessions of FMR therapy with four-week intervals. One hour before the laser therapy, topical anesthesia (EMLA, Astra-Zeneca, Sodertalje, Sweden) was applied to the lesions. As a safety measure, the patients and the dermatologist used safety goggles whenever FMR + CO_2_ procedure was performed.

The characteristics and settings of the FMR + CO_2_ laser equipment (Qray FRX, DOSIS, Germany) were as follows: laser type: ultra pulse, 10600 nm; laser power: 16 ± 2 J/cm^2^; laser energy: 20-, 30 millijoules; ablation depth: 400–600 micrometers; dot cycle (duration): 5 ± 2 milliseconds; and pixel pitch: 0.8 ± 0.1. In each session, two laser pulses were delivered.

In addition, settings of the FMR device (INFINI*™*, Lutronic, Goyang, Korea) were as follows: depth: 1.5–3 mm; level: 5–9; and time: 110–150 ms. In each session, three laser pulses were delivered.

In each session, after laser therapy, the patients were advised to clean the lesions by normal saline solution and cover it with sterile Vaseline gauze for 24 h. In addition, mupirocin and zinc oxide ointments were applied for two days.

### 2.3. Measurements and Evaluations

Assessments were made by photographing all the striae in both groups by a digital camera (Canon Power Shot SX260 HS) at the baseline, one month, and three months. The surface area of the striae (mm^2^) was measured by PictZar Digital Planimetry Software (Ver. 5.05.2, Biovisual Technologies, New Jersey, USA). Photographs of pre- and posttreatment were evaluated by two dermatologists who were blinded to the type of treatments. Finally, the percentage of improvement was assessed using the following four-point scale: 0%–25% improvement: weak, 25%–50% improvement: moderate, 50%–75% improvement: good, and more than 75% improvement: excellent [21]. The primary overall efficacy was considered when the improvement was more than 50%.

In addition, the patients were asked to provide their opinions about improvements in each treatment group using the patient satisfaction Visual Analog Scale (VAS). Patient satisfaction VAS is a self-administered 10-point scale; 0 represents lack of improvement and 10 in the scale indicates complete improvement [21].

Patients were assessed at two-week intervals for possible side effects such as infection, erythema, bleeding, pain, burn, ulcer, scar, and keloid formation. During this time, the postinflammatory pigmentation of the striae was assessed. Three months after the last session of treatment, all patients were followed up for possible side effects.

All statistical analyses were performed by Statistical Package of Social Sciences (SPSS) version 19.0. Paired *t*-test, Wilcoxon signed-rank test, repeated measure analysis, and Chi-square test were used. A *p* value of <0.05 was considered significant.

## 3. Results

The characteristics of the six patients involved in this study and baseline values are summarized in Table 1.

The primary overall efficacy was 72.9% and 75% among the FMR + CO_2_ group and 47.9% and 50% among FMR group in the first and second follow-up, respectively.

**Figure 2:**
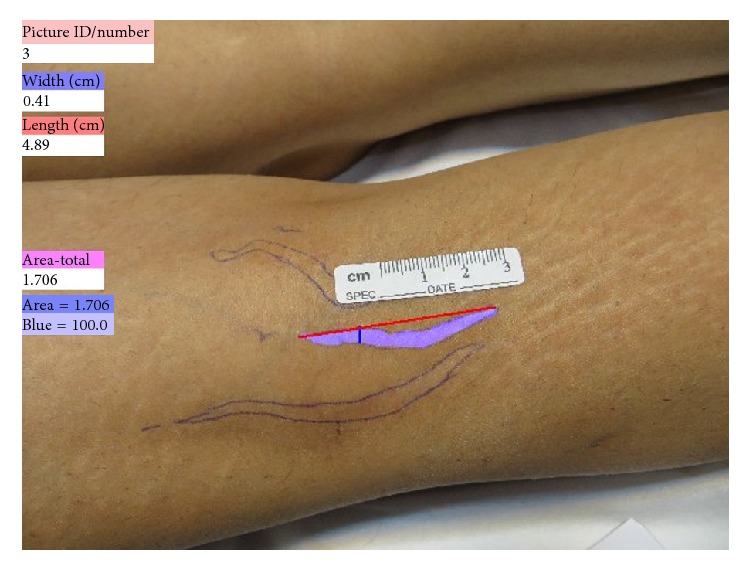
Striae alba on the right calve one month after the end of study. The purple areas denote the measured area by PictZar Digital Planimetry Software.

The mean surface area of the striae before treatment was 257.43 ± 161.75 mm^2^ in the FMR + CO_2_ group and 259.05 ± 159.79 mm^2^ in the FMR group (Figure 2).

No significant difference was found between the two groups using the paired *t*-test (*p* = 0.421). The mean surface area of the striae, after treatment, was significantly higher in the FMR group than in the FMR + CO_2_ group in the first follow-up (*p* = 0.001) and the second follow-up (*p* = 0.001) (Table 2). Repeated measure analysis revealed that the mean surface area of the lesions significantly decreased in both groups after the treatment [FMR + CO_2_ group: *F*(1,47) = 157.18, *p* < 0.001; FMR group: *F*(1,47) = 99.95, *p* < 0.001] (Table 2).

**Figure 3:**
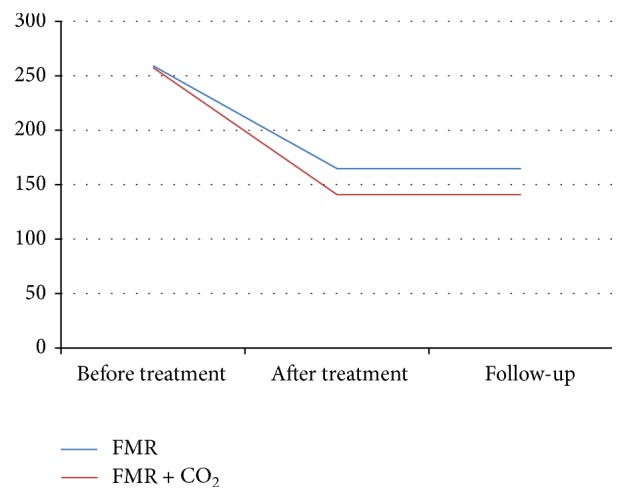
The measured SA area at baseline, after treatment and in the follow-up in FMR and FMR + CO_2_ group.

Figure 3 shows the measured SA area at the baseline and after treatment in the first and second follow-up in both groups (Figure 3).

**Figure 4:**
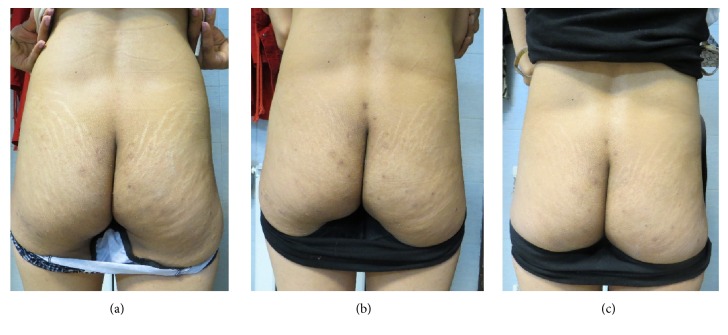
Striae distensae on the buttock of a 30-year-old patient. Significant improvement at 3 months after the end of the study: (a) at baseline; (b) after one month; (c) after three months.

**Figure 5:**
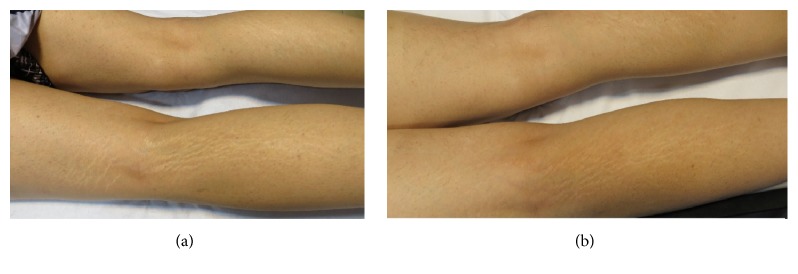
Improvement in clinical appearance of striae alba. Baseline (a) and posttreatment (b) with fractionated microneedle radiofrequency combined with fractional carbon dioxide laser.

Wilcoxon signed-rank test showed significantly higher clinical improvement in the FMR + CO_2_ group than in the FMR group in the first follow-up (median = 3 and 2, resp.; *p* = 0.002) and the second follow-up (median = 3 and 2, resp.; *p* = 0.004) (Figures 4 and 5).

In addition, there was a significant difference between the two treatments in patient satisfaction VAS scores in the first and second follow-up visits; better results were reported in the FMR + CO_2_ group than in the FMR group (*p* < 0.001) (Table 3).

**Figure 6:**
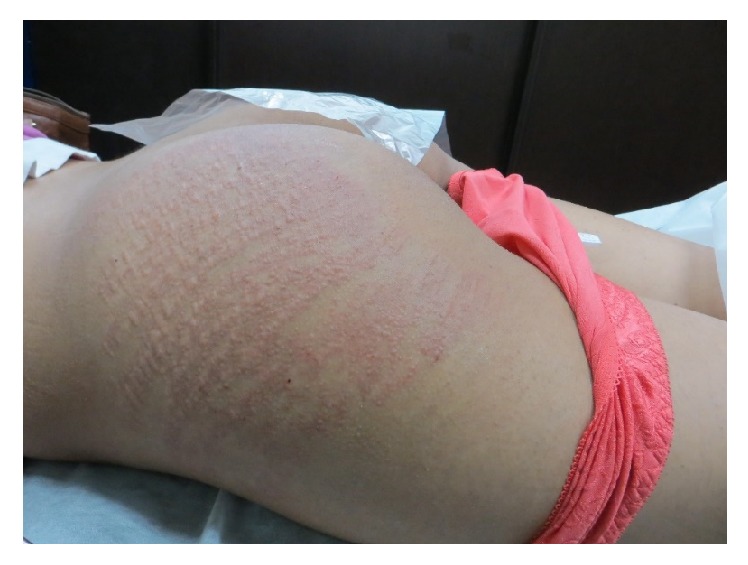
Immediate erythema at the site of treatment in a patient in FMR + CO_2_ group.

All patients experienced erythema in both groups (Figure 6); however, it was relieved within two weeks after intervention. In FMR + CO_2_ group, 47.9% of patients felt slight pain and 52.1% felt moderate pain; these figures were 43.8% and 56.3% in the FMR group, respectively. However, this difference was not significant between the two groups (*p* = 0.682). No significant difference was observed in the presence of edema between the two groups (*p* = 0.601).

Transient postinflammatory hyperpigmentation (PIH) occurred in 9 of the 48 SD in FMR + CO_2_ group and was complete resolution spontaneously for 3 months. None of the patients in the other group experienced any PIH. So there was a significant difference between the two groups with respect to occurrence of PIH that was more significant in FMR + CO_2_ group (*p* = 0.004).

None of the patients experienced any infection, ulcer, burn, or scar.

## 4. Discussion

The results of this study showed that FMR + CO_2_ laser therapy caused a greater reduction in the surface area of the lesions, with a higher patient and dermatologist satisfaction.

Although many studies have been conducted on SD, a standard treatment has not yet been found. Topical therapies do not provide satisfying results [22]. In recent years, laser therapy and light devices have become popular such as pulsed-dye laser (PDL) [23], copper bromide laser [24], excimer laser [25], intense pulsed light [26, 27], 1,064 nm Nd: YAG laser [28], fractional nonablative 1540 nm laser [4], and fractional photothermolysis [8, 10, 29]. However, these modalities usually have a poor effect on SA [3]. Among these, fractional photothermolysis is suggested as an effective treatment for SA [8, 10, 30]; however, the results are unpredictable. Hence, new modalities for improving the treatment of SA are much required.

In 2013, Ryu et al. introduced a new method of laser therapy using the FMR + CO_2_ laser with positive results among Korean patients [17]. In that study, 30 females (mean age of 33 years with skin type IV) with SA were allocated to the fractionated CO_2_ alone, FMR alone, or the combination FMR + CO_2_ laser group. The mean clinical improvement score was 2.2, 1.8, and 3.4 in fractionated CO_2_ laser group, FMR group, and combination group, respectively [17]. Our results are consistent with those of this previous study [9]. In Ryu's study, thickening of the epidermis, increased number of collagen fibers, a high expression of TGF-*β*1, and stratifin in the combination group were observed. Yet, it was concluded that the combination of FMR + CO_2_ laser was a good alternative treatment for SA. Our improvement score in the FMR group was similar to that in [17] on the other hand, our improvement score in FMR + CO_2_ group was less similar. These differences can be justified by the use of different laser therapy devices made by different companies, different device settings, and patients with different races.

In addition, the present study included participants with skin type III, whereas Ryu's study included participants with skin type IV [17]. Even the laser therapy sessions were different: our study included three sessions of FMR therapy and two sessions of FMR + CO_2_ laser therapy, while Ryu's study included three sessions of FMR therapy and three sessions of the combination therapy [17].

A study by Naeini and Soghrati examined 92 SA lesions for fractional CO_2_ laser treatment or 10% glycolic acid + 0.05% tretinoin cream therapy in Iranian patients [31]. Their results showed that, in comparison with topical treatment (−7.9 ± 9 cm^2^), the mean difference of the striae surface area before and one month after treatment in fractional CO_2_ laser decreased significantly (−37.1 ± 15.6 cm^2^). The mean VAS score was also significantly higher in the laser therapy group (3.05 ± 0.74) than in topical treatment group (0.63 ± 0.66). Nikyar et al. also evaluated the efficacy of fractional CO_2_ laser alone and in combination with PDL for SA lesions [9]. In 88 lesions, the mean surface area difference before and after treatment decreased in the combined group. Similarly, the improvement was better for VAS and dermatologist-assessed improvement scale at one month after treatment in the combined group. These two studies confirm the effectiveness of fractional CO_2_ laser therapy among Iranian patients, which is consistent with our results. However, the combination method in our study is more effective from the dermatologist's point of view and for greater patient satisfaction. Furthermore, our method was more cost-effective than that of the other two studies.

Yang and Lee were able to conclude that the disk microneedle therapy system was a safe and effective treatment for SD in Korean patients [14].

The mean improvement score after three sessions of treatment was 2.4 out of a four-point scale. Of 18 patients, half of the patients were satisfied, with six patients being highly satisfied. However, a different race was involved and the study was conducted in males; furthermore, SD included patients with striae rubra and SA.

There are some limitations to our study. Firstly, all participants were females and the sample size was not too large. Secondly, a skin biopsy was not performed because of noncooperation from the patients.

## 5. Conclusions

An appropriate treatment for a disease should be safe, effective, and cost beneficial. There are several different treatments for SA from topical to laser therapies; however, most of them are not effective. Combination treatments have proven to be safe and effective.

This study showed that FMR in combination with fractional CO_2_ laser had more therapeutic effect on SA than FMR alone, without serious side effects. However, more studies using other modalities are encouraged to find more effective therapies for the management of SA.

## Figures and Tables

**Table 1:** Patients' characteristics.

Variable	Mean ± standard deviation/frequency (%)
Age	30.17 ± 5.19
Sex	
Female	6 (100%)
Male	0 (0%)
Body mass index	21.43 ± 1.31
Family history	
Positive	5 (83%)
Negative	1 (17%)
Cause	
Weight gain	6 (100%)
Type of skin	
Type III	6 (100%)
Duration (years)	10.83 ± 1.33

**Table 2:** Mean surface area before treatment and after treatment in FMR + CO_2_ and FMR-treated groups.

Surface area	FMR + CO_2_	FMR	*p* value^*∗∗*^
Before treatment	257.43 ± 161.75^*∗*^	259.05 ± 159.79	0.421
After treatment	140.92 ± 133.62	164.67 ± 124.63	0.001
*p* value^*∗∗∗*^	<0.001	<0.001	

^*∗*^Mean ± standard deviation, ^*∗∗*^paired sample *t*-test, and ^*∗∗∗*^repeated measure analysis.

**Table 3:** Mean of patient satisfaction VAS score after treatment and in the follow-up in both groups.

	FMR + CO_2_	FMR	*p* value^*∗*^
VAS score after treatment	7.08 ± 1.03	5.56 ± 0.99	<0.001
VAS score in the follow-up	7.12 ± 1	5.60 ± 0.96	<0.001
*p* value^*∗*^	0.159	0.322	

^*∗*^Paired sample *t*-test.
